# The Development of the SARS-CoV-2 Epidemic in Different Regions of Siberia in the 2020–2022 Period

**DOI:** 10.3390/v15102014

**Published:** 2023-09-27

**Authors:** Natalia V. Palyanova, Ivan A. Sobolev, Andrey Yu. Palyanov, Olga G. Kurskaya, Andrey B. Komissarov, Daria M. Danilenko, Artem V. Fadeev, Alexander M. Shestopalov

**Affiliations:** 1 Federal Research Center of Fundamental and Translational Medicine, 630117 Novosibirsk, Russia; sobolev_i@hotmail.com (I.A.S.); palyanov@iis.nsk.su (A.Y.P.); kurskaya_og@mail.ru (O.G.K.); shestopalov2@mail.ru (A.M.S.); 2 A.P. Ershov Institute of Informatics Systems, Siberian Branch of the Russian Academy of Sciences, 630090 Novosibirsk, Russia; 3 Department of Mathematics and Mechanics, Novosibirsk State University, 630090 Novosibirsk, Russia; 4 Federal Budgetary Institution «Smorodintsev Research Institute of Influenza», 197376 St. Petersburg, Russia; andrey.komissarov@influenza.spb.ru (A.B.K.); daria.baibus@gmail.com (D.M.D.); afadeew@gmail.com (A.V.F.)

**Keywords:** SARS-CoV-2, COVID-19, epidemiology, phylogenetics, Siberia, recombinants

## Abstract

The comparison of the development of the SARS-CoV-2 epidemic in several neighboring regions can help researchers to assess the risks and develop more effective strategies and approaches in the field of preventive medicine. We analyzed the infection and mortality statistics for the 2020–2022 period in ten individual regions of the Siberian Federal District of Russia. We also sequenced complete genomes, which allowed us to analyze the genetic diversity of SARS-CoV-2 circulated in each of the ten regions and to build a phylogenetic dendrogram for the virus variants. The ParSeq v.1.0 software was developed to automate and speed up the processing and analysis of viral genomes. At the beginning of the pandemic, in the first two waves, the B.1.1 variant (20B) dominated in all regions of the Siberian Federal District. The third and fourth waves were caused by the Delta variant. Mortality during this period was at a maximum; the incidence was quite high, but the number of deposited genomes with GISAID during this period was extremely low. The maximum incidence was at the beginning of 2022, which corresponds to the arrival of the Omicron variant in the region. The BA.5.2 variant became the dominant one. In addition, by using NextClade, we identified three recombinants in the most densely populated areas.

## 1. Introduction

The first outbreak of a new severe acute respiratory syndrome coronavirus was reported in Wuhan, China, in December 2019; later, the virus spread widely around the world, and on 11 March 2020, the WHO declared a pandemic of SARS-CoV-2, which causes the respiratory disease COVID-19 [1]. The first two cases of the disease in Russia were detected in January 2020, but it did not give rise to a pandemic at that moment. Thus, the outbreak in Russia only started on 2 March 2020 in Moscow, which was later than in many European countries [2]. Here, we analyze the emergence of SARS-CoV-2 in the Siberian Federal District of Russia in the 2020–2022 period. The region is located in the middle of Eurasia, and it has extensive transport links with Russian cities and most countries in Europe and Asia. The area of the region is 4.36 million square kilometers and the population density is 3.9 people per square kilometer [3]. The first confirmed case of SARS-CoV-2 in the Siberian Federal District was on 14 March 2020 [4].

Already, by the beginning of 2020, SARS-CoV-2 proved to be a dangerous pathogen of acute respiratory viral infections with an unpredictable course and a high mortality, affecting various organs both as a result of direct infection and due to the excessive activation of the body’s immune response [1]. The virus spreads through contact with infected people via the air, as particles are sprayed into the air when coughing, sneezing or talking, and it is also transmitted through touching the mucous membranes with hands that have touched surfaces with the virus on them [5].

The epidemic process of COVID-19 has its own characteristics in each country and in each region of Russia [2,6]. The development of the pandemic in Russia is described for several regions, but it is hard to compare them since all articles cover different epidemiological and temporal aspects of the pandemic. The epidemiologic features of COVID-19 were described for Yakutia [7], the Lipetsk region [8] and the northwest of Russia [9]. The spread of the infection is influenced by important parameters of the region such as the age structure, the population density and size, transport links with other regions and countries, cultural characteristics, access to medical care and environmental factors [10,11,12]. A retrospective analysis of the incidence and herd immunity in Russia [6,13,14] helps to evaluate the effectiveness of restrictive measures to plan future activities aimed at containing epidemics of acute respiratory infections with pandemic potential [12]. Even with a low mortality, the main problem is the high burden on healthcare systems. Strategies to prevent a rapid rise in cases include social distancing, wearing masks, washing hands and using antiseptics, limiting mass gatherings, introducing non-working days and introducing distance learning in schools and universities [10,11]. A phylogenetic analysis showed multiple importations of the virus into Russia [15].

In our previous work, we analyzed the beginning of the pandemic in the Novosibirsk region [16]. At the initial stage of the pandemic in the Novosibirsk region on 1 April 2020, in our laboratory, 13,699 samples of biological material were collected from people who showed symptoms of acute respiratory illness and who had contact with patients with a confirmed diagnosis of COVID-19, from people who crossed the Russian border and from healthcare professionals. We analyzed the questionnaires and corresponding samples and described the incidence pattern according to sex and age, and identified a significant number (42%) of asymptomatic carriers. In addition, 15 samples were selected for sequencing and a subsequent phylogenetic analysis, which showed that at the beginning of the pandemic in the Novosibirsk region, there were already variants belonging to the clades B.1, B.1.1 and B.1.1.129. The article also provides an analysis of the amino acid substitutions in the sequenced samples. In this paper, we continue and expand this study from the Novosibirsk region to the Siberian Federal District. In the example of the ten regions of the Siberian Federal District, we analyze the development of the SARS-CoV-2 epidemiological situation during the 2020–2022 period. In this work, we combine the incidence and demographic data with phylogenetic approaches, which have helped to track the virus evolution and genetic changes and inform public health strategies and epidemiological concepts.

## 2. Materials and Methods

### 2.1. Collecting Epidemiological Data in the Siberian Federal District

**Figure 1:**
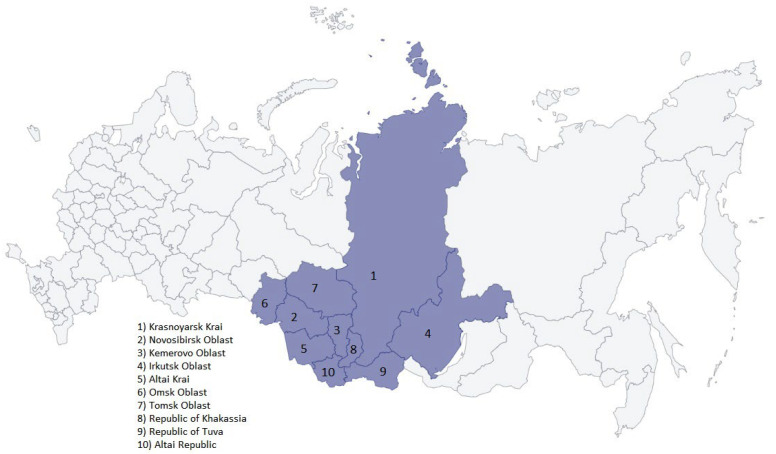
The map of the Siberian Federal District in Russia.

In this paper, we analyze the data for the number of detected cases of SARS-CoV-2 in ten Siberian regions, obtained from the official website: https://стoпкoрoнавирус.рф (accessed on 9 August 2023) [4]. All aspects of the study were approved by the Ethics Committee of the Federal State Budget Scientific Institution “Federal Research Center of Fundamental and Translational Medicine”. Written informed consent was obtained from all of the tested people, or their parents or official representatives, prior to sample collection. For analysis, the collected data included the absolute numbers of cases and deaths per day in each region of the Siberian Federal District: Krasnoyarsk Krai, Novosibirsk Oblast, Kemerovo Oblast, Irkutsk Oblast, Altai Krai, Omsk Oblast, Tomsk Oblast, the Republic of Khakassia, the Republic of Tuva and the Altai Republic (Figure 1 and Table 1).

The population data were taken from the https://rosstat.gov.ru/ official site (accessed on 9 August 2023) [17]. The population density and the map (Figure 1) were taken from http://sfo.gov.ru/okrug/ (accessed on 9 August 2023) [3]. The simple moving average method was used to smooth the graphs. Each date corresponds to a seven-day average (data for that day, three days before and three days after that date). For example, the data from dates from 1 to 7 are summed, divided by 7, and recorded for date 4. The entire observation period (2020–2022) was divided into periods corresponding to the incidence waves. The beginning of each wave corresponds to the beginning of the rise in the incidence; the end of the wave corresponds to the minimum incidence between adjacent waves.

### 2.2. The Next-Generation Sequensing

The nucleotide sequences of the SARS-CoV-2 variants circulated in the Siberian Federal District in the 2020–2022 period were obtained by next-generation sequencing (NGS) in the WHO National Influenza Centre (Research Institute of Influenza) and the Federal Research Center of Fundamental and Translational Medicine [16], and most of them were deposited in the GISAID database. The selection of samples for sequencing was based on obtaining from several healthcare institutions located in different regions of the Siberian Federal District.

The complete genome NGS sequencing was performed using the Illumina MiSeq platform and the associated reagent kits (Illumina), according to the manufacturer’s methodology (Illumina, Inc. Worldwide Headquarters: 5200 Illumina Way, San Diego, CA 92122, USA). RNA was extracted using the QIAamp Viral RNA Mini Kit (Qiagen, Hilden, Germany). Whole-genome amplification was performed using the ARTIC primer set [18]. DNA libraries were prepared using a Nextera DNA Flex Library Prep kit (Illumina, San Diego, CA, USA). Sequencing of the DNA libraries was conducted on a MiSeq genome sequencer (Illumina) with a reagent kit, version 3 (600-cycle). The consensus sequences were generated using Bowtie 2 software [19].

### 2.3. The Analysis of Genetic Data

The analysis of genome-wide nucleotide sequences for belonging to a phylogenetic clade according to the PANGOLIN classification [20], as well as the identification of recombinants, was carried out using NextClade [21]. The multiple alignment was also performed using the NextClade v2.14.1 online [22].

For visualization in the phylogenetic dendrogram, a selection of sequences was made: for each region, 1–2 representatives of each clade found in this region were selected, usually the earliest and sometimes the latest (if there was any). There were only two sequences from Tomsk, so we included one of them despite their low quality.

We also included three recombinants despite the low quality. The recombinant variant is the mutant hybrid of two sublineages [23]. For example, a hybrid combination of the Delta (AY.4) and BA.1 Omicron variants has been nicknamed “Deltacron” [23].

We developed the accessory program named ParSeq v.1.0. (Parser of Sequences) for convenient access to metadata, filtering by various criteria, sorting, analyzing and editing sequences). The program is quite simple and has no convenient user interface at the moment. A significant expansion of the functionality of the program (including the online version with a web interface) is planned for the near future and will be published in a separate article. Duplicate genomes that had the same dates, locations and nucleotide sequences were removed using the ParSeq v.1.0 program, low quality sequences (too many nucleotides that were not determined by sequencing, ‘N’-s) were excluded. And editing such as removing untranslated regions, 5′UTR and 3′UTR, was also performed using the same software.

For the earliest sequences corresponding to the most common clades, reference sequences were determined: for each selected sequence, ParSeq v.1.0 ran NCBI BLASTN 2.14.0 + x64 (locally installed) [24] on subsamples from GISAID and Genbank, including sequences from both databases, received no later than the date of the analyzed sequence. The one that had the least number of differences with the analyzed sample (the total number of point mutations, substitutions and/or insertions) was chosen as a reference. The result was automatically extracted by the ParSeq v.1.0 program from the output file created by BLASTN based on the results of its work.

Thus, in order to build a phylogenetic dendrogram, the nucleotide sequences determined by sequencing, the closely related sequences determined by BLAST analysis, and the reference sequence from Wuhan were included in the general multiple alignment.

Phylogenetic analyses were performed with MEGA11 [25] software using the maximum likelihood method (Maximum Likelihood) with bootstrap support (500 replications) and utilizing a generalized time-reversible substitution model with a gamma-distributed rate variation across sites and a proportion of invariant sites (“GTR + G + I”) [26]. The visualization of the phylogenetic tree was also performed with MEGA11.

## 3. Results

### 3.1. Detection of SARS-CoV-2 in the Siberian Federal District

To analyze the differences in the epidemiological process, Table 1 presents data on the size and density of the population of the regions of the Siberian Federal District, the number of infections, deaths, mortality, as well as the maximum number of infections and deaths in one day. The maximum number of infected and dead per day reflects the peak load, and relative to the population—the effectiveness of the strategy to smooth out the peaks of infection. In those regions where the number of infected and dead per day in relation to the population is high, smoothing was not effective enough (Table 1). If the peak was not high due to the longer wave of infections, then the peak load on the healthcare system was reduced. According to Table 1, the population density does not affect the incidence and mortality.

**Figure 2:**
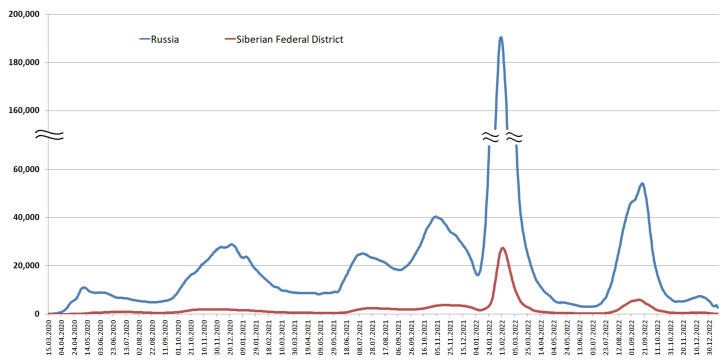
Moving average number of SARS-CoV-2 infections detected per day in the Siberian Federal District and in Russia. Blue—averaged data for the whole of Russia, red—Siberian Federal District.

Due to the remoteness of the region and the measures introduced to prevent the spread of SARS-CoV-2, the epidemic in the Siberian Federal District developed later than in Russia as a whole (Figure 2).

The beginning of the first wave was approximately in the same days in all regions of the Siberian Federal District and 17 days later than in Moscow. The peak of the first wave was in 2–12 May in Moscow and in 8–14 July in the Siberian Federal District. In addition, it turned out that in the Siberian Federal District the duration of the first wave is much longer, and the rate of increase in the incidence is much less than in Moscow, which indicates a significant smoothing of the peak in the first wave in Siberia.

**Figure 3:**
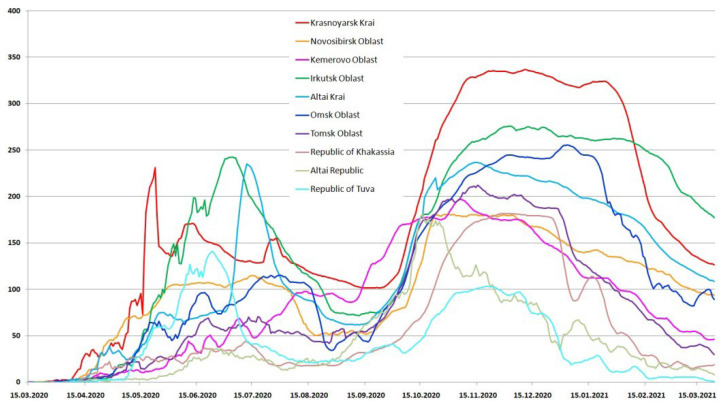
Daily number of SARS-CoV-2 cases according to official data in the Siberian Federal District during the first and second waves (7 day moving average).

The Krasnoyarsk Krai is the largest region of the district, both in terms of population and area due to the northern sparsely populated territories, which makes the population distribution heterogeneous and makes it difficult to analyze the relationship between population density and the epidemiological situation. The largest number of cases and deaths in the Siberian Federal District is in the Krasnoyarsk Krai, which corresponds to the maximum population and shows that population density affects these indicators less. The populations of the Novosibirsk, Kemerovo and Irkutsk regions are close to the Krasnoyarsk Krai, but the population density varies significantly. The Irkutsk region was among the leaders in terms of incidence in both the first and second waves, yielding only to the Krasnoyarsk Krai in the second wave. While in the most densely populated Kemerovo region, at the beginning of the pandemic, there was a gradual slow increase in the number of infected people, the first wave lasted almost a year, while in other regions of the Siberian Federal District two waves of infection are clearly distinguished during the same time (Figure 2). Figure 3 shows the daily detection of SARS-CoV-2 cases in the Siberian Federal District during the first and second waves.

Such an effective smoothing of the infection curve led to low mortality in the region, and the maximum number of deaths in one day was 12, in contrast to the Krasnoyarsk Krai, Irkutsk Oblast and Altai Krai, where the maximum number of deaths in one day exceeded 30 people (Table 1). Effective measures to prevent the spread of a new coronavirus infection were taken in the Novosibirsk Oblast. Despite the rather high population density and size, the peak of the first wave was significantly smoothed out and turned out to be lower in absolute terms not only in comparison with such large population regions as the Krasnoyarsk Krai and the Irkutsk Oblast, but also in comparison with the less densely populated Tuva and Altai Krai. The low rate of the maximum number of deaths per day (18) and low mortality also confirms the effectiveness of the incidence waves smoothing.

The two least populated regions, the republics of Tuva and Altai, have similar population densities and numbers of people, but the epidemiological process has been different. In Tuva, a rather sharp and high rise in the incidence was observed in the first wave. At the peak of the first wave, Tuva overtook such densely populated areas as the Novosibirsk, Omsk and Tomsk regions, as well as Khakassia, in terms of the number of infected people. At the same time, the second wave was even smaller than the first, while in other regions the second wave was larger or comparable (Figure 3).

**Figure 4:**
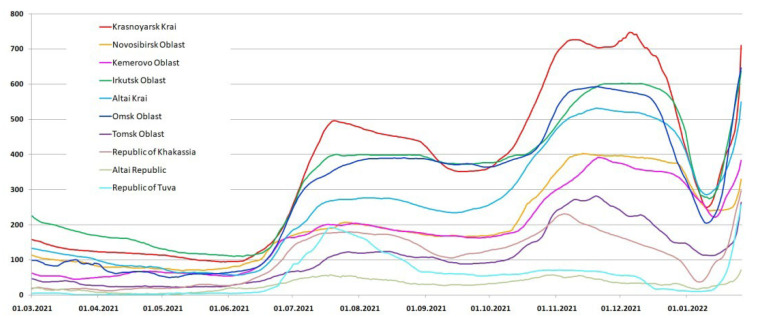
The daily number of people infected with SARS-CoV-2, according to official data in the Siberian Federal District, in the third wave, corresponding to the arrival of the Delta variant in Russia, smoothly flowing into the fourth wave (7 day moving average).

**Figure 5:**
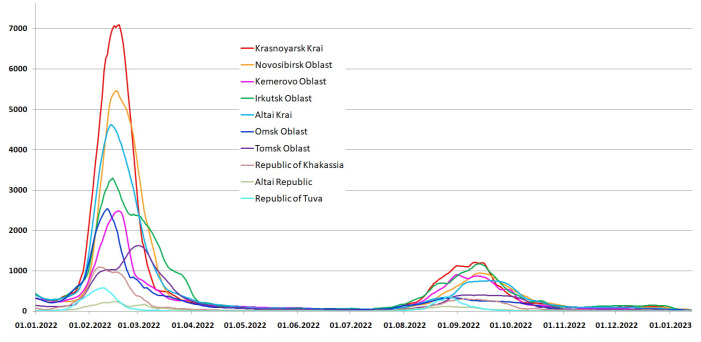
The daily number of people infected with SARS-CoV-2 according to official data in the Siberian Federal District in the fifth and sixth waves of incidence (7 day moving average).

The epidemic developed most unpredictably in the Altai Krai. The first wave began with a quick but short rise, followed by a decline and another sharp rise to a plateau. However, after the plateau, instead of a decline, another sharp increase began, thus, the peak of the first wave in the Altai Krai turned out to be the later than in other regions and one of the highest (with the Krasnoyarsk Krai and the Irkutsk Oblast). During subsequent waves (Figure 4 and Figure 5), the trend towards higher incidence than it could be expected based on the size and density of the population, continued, which led to the highest final mortality compared to other regions of the Siberian Federal District (Table 1).

After the delay of the second wave, a simultaneous rise of the incidence began in all regions of the Siberian Federal District (Figure 4). A new increase in the incidence corresponds to the arrival in the region of the Delta variant, which dominated during this period in Russia. The incidence dynamics was similar in all regions of the Siberian Federal District; however, the amplitude did not always correspond to the population size. Thus, in the Altai Krai, Irkutsk Oblast and Omsk Oblast, the incidence in absolute values was higher than in the Novosibirsk Oblast and Kemerovo Oblast, where the population size and density are higher. This confirms the effectiveness of anti-epidemic measures taken in these regions.

A slight decrease in the incidence at the end of the third wave was observed only in the Krasnoyarsk Krai and in Tuva, and then unlike in other regions of the Siberian Federal District, there was no fourth wave in Tuva and the Altai Republic. In all other regions of the Siberian Federal District, immediately after the plateau of the third wave, a new increase in the incidence began in the middle of September 2021. However, we cannot associate this rise with the arrival of the Omicron variant, since it appeared in Russia only at the end of December 2021 [27,28]. Most likely, the rise after the start of the decrease is associated with the beginning of the school year. In most regions, masks or distance learning for schoolchildren were not announced, which led to an explosive increase in the number of cases (Figure 4).

Despite the significant decrease in the official incidence in early January 2022, the real incidence most likely did not fall. The first 10 days of January were national holidays, so most of the labs were closed, which looks like a drop in the number of infections at the beginning of the year. Active social contacts during the New Year holidays without social distancing led to a huge outbreak at the beginning of 2022, which looked like a fifth wave, which began in the Tuva and the Altai Republic much later than in the other regions of the Siberian Federal District. The sharp simultaneous rise in the incidence and the almost simultaneous end of the fifth “Omicron” wave ended the exacting period—the incidence for the first time fell to the level of the beginning of the pandemic (Figure 5).

The sixth wave began simultaneously in all regions of the Siberian Federal District in early August 2022. Due to the escalation of the epidemiological situation, on 12 August, the mandatory mask regime was returned in Tuva, and by September, masks in public places became mandatory in the Altai Republic. Figure 5 shows that in these two regions, the sixth wave turned out to be shorter, which confirms the effectiveness of the mask regime. The maximum number of detected cases of SARS-CoV-2 in all subjects of the Russian Federation occurred during the circulation of the Omicron variant.

### 3.2. COVID-19 Mortality in the Siberian Federal District

**Figure 6:**
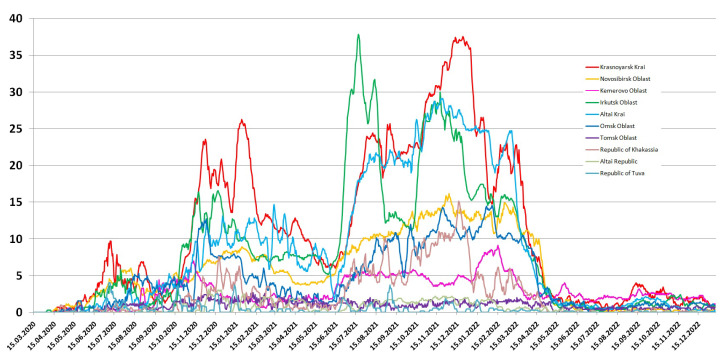
Daily COVID-19 mortality in the Siberian Federal District in 2020–2022 (7 day moving average).

One of the indicators of the epidemiological process is the dynamics of mortality. Figure 6 shows the moving average for daily COVID-19 mortality in the Siberian Federal District. Mortality, related to the number of cases, reflects not only the lethality of the infection, but also indirectly reflects the burden on the healthcare system. The maximum mortality occurred at the beginning and middle of the pandemic, when the most pathogenic variants of the virus were circulating. In Russia [27,28], as well as throughout the world [29], mortality was low in the first wave of incidence, and it was maximal in the third and fourth waves with the AY.122 dominating variant, and minimal during circulation of exclusively the Omicron genovariants.

In contrast to incidence, mortality in all regions of the Siberian Federal District, except for the Irkutsk region, looks like three waves, not six. The first two waves of mortality correspond to the first two waves of morbidity. Since death from COVID-19 can occur within a month or more [1] from the onset of the disease, a decrease in incidence does not lead to a sharp decrease in mortality, and if the next wave occurs quickly enough, then mortality simply does not have time to drop. Thus, the mortality from the third, fourth and fifth waves merged into one big wave with a peak between the Delta and the Omicron (just when the incidence was minimal). Four waves of mortality are observed in the Irkutsk region, although the timing of the incidence does not differ from neighboring regions.

The high death rate corresponding to the fifth wave is due, firstly, to the ongoing circulation of the Delta, and secondly, to a very high number of cases, which led to an overload of the healthcare system. Mortality corresponding to the sixth wave of incidence in all regions turned out to be significantly lower than during the remaining five waves, which is consistent with the described low lethality of Omicron [30].

For the entire period of observation, the maximum mortality was observed in the Krasnoyarsk Krai, which corresponds to the maximum incidence (Figure 6). The duration of the wave of increased mortality significantly exceeds the duration of the wave of morbidity, because a death from COVID-19 can also occur quite a long time after infection. The highest lethality of SARS-CoV-2 was recorded in the Altai Krai (2.8%), and the increased lethality was also in the Irkutsk Oblast (2.42%), Krasnoyarsk Krai (2.63%) and Khakassia (2.35%). The increase in mortality in these regions was sharper, slightly lagging behind the rising incidence, suggesting that the health care system may have been overwhelmed and medical assistance was not provided in time. Also in the Irkutsk Oblast, there were two sharp peaks in mortality from June 2021 to April 2022, while in other regions at this time there was a uniform change in mortality. The short period of low mortality from COVID-19 during the period of activity of the Delta variant may be a consequence of the delayed registration of deaths due to the overload of the healthcare system, since there was no decrease in the incidence during this period. On the contrary, in the Tomsk Oblast and Tuva, the mortality rate remained at approximately the same low level, and as a result, the mortality rate for the entire period of the pandemic turned out to be a record low—0.61% and 0.68%.

### 3.3. The Diversity of Genetic Variants of SARS-CoV-2 in the Siberian Federal District

We analyzed 1988 genome-wide SARS-CoV-2 sequences obtained by NGS sequencing in the Siberian Federal District in 2020–2022. After the operation of removing duplicates, 1889 sequences were suitable for analysis. Where possible, only high quality sequences were used, but for the period corresponding to wave 5, only four good quality sequences and nine low quality sequences were found, so it was decided to include all of them in the initial analysis.

We revealed an irregular distribution of the number of sequences obtained in different times and from different regions of the Siberian Federal District. Thus, a total of 230 sequences were obtained in 2020, 37 corresponding to 2021 and 1622 obtained in 2022. The largest number of sequences was obtained from Novosibirsk (883), Kemerovo (384) and Omsk (275), and the smallest number of sequences was from Republic of Tuva (0) and the Altai Republic (0). The analysis of the incidence in the Republic of Tuva and the Altai Republic shows that the epidemiological process proceeded there differently than in other regions of the Siberian Federal District. Phylogenetic analysis would help to distinguish the difference between the virus variants in these regions and those in the remaining regions of the Siberian Federal District, but these are the least populated regions, and not a single sequence has been obtained from there during the entire pandemic.

The diversity of virus variants circulated in Siberian Federal District, according to NextClade [21] analysis of genome-wide sequences, is shown in Table 2. 

In the first and the second wave, the dominant genetic variant of SARS-CoV-2 circulated in the Siberian Federal District was B.1.1 (20B). During the first wave, B.1 (20A), B.1.1.317 (20B), B.1.1.294 (20B), B.1.1.348 (20B), B.1.1.163 (20B), B.1.1.83 (20B), and B.1.1.141 (20B) were also found. During the second wave, B.1.1.141 (20B), B.1.1.294 (20B), B.1.1.317 (20B), B.1.1.348 (20B), B.1.1.397 (20B), B.1.1.398 (20B), B.1.1.525 (20B), B.1.1.523 (20B), AT.1 (20B) and B.1.1.7 (20I, Alpha variant) were also found.

The third wave corresponded to the appearance of the Delta variant in the Siberian Federal District. In addition to the Delta (AY.122), variant B.1.1 (20B) was still detected; however, the small number of sequences for this period does not allow us to say what other variants could have circulated at that time. The fourth wave, according to our data, was completely caused by the Delta variant, which is in good agreement with the situation in Russia during this period [27,28].

The fifth wave corresponded to the arrival of the BA.1.1 (21K) Omicron variant, which had previously dominated in the European part of Russia, although the AY.126 (21J) and AY.122 (21J) variants still continued to circulate. The incidence in the fifth wave was incredibly high (Figure 5), but very few sequences were obtained during this period. On the contrary, in the sixth wave, the incidence was at the level of the third and fourth waves, but the number of sequences turned out to be large, which allowed us to identify the diversity of omicron variants circulating in the Siberian Federal District at the end of 2022. The most numerous and widespread variant was BA.5.2 (22B), and 69 more variants of the virus were identified (Table 2).

Although the earliest Omicron sequence in Russia was identified at the end of 2021 [28], the Delta continued to circulate for quite a long time. Since 24 June 2022, we have not identified a single Delta strain or other earlier variants. We believe that from this moment the sixth wave of incidence, corresponding completely to the Omicron variant, begins.

In addition, using NextClade [21], we identified three recombinants (Table 3), which were collected in the most densely populated areas.

### 3.4. The Phylogenetic Tree of Genetic Variants of SARS-CoV-2 in the Siberian Federal District

**Figure 7:**
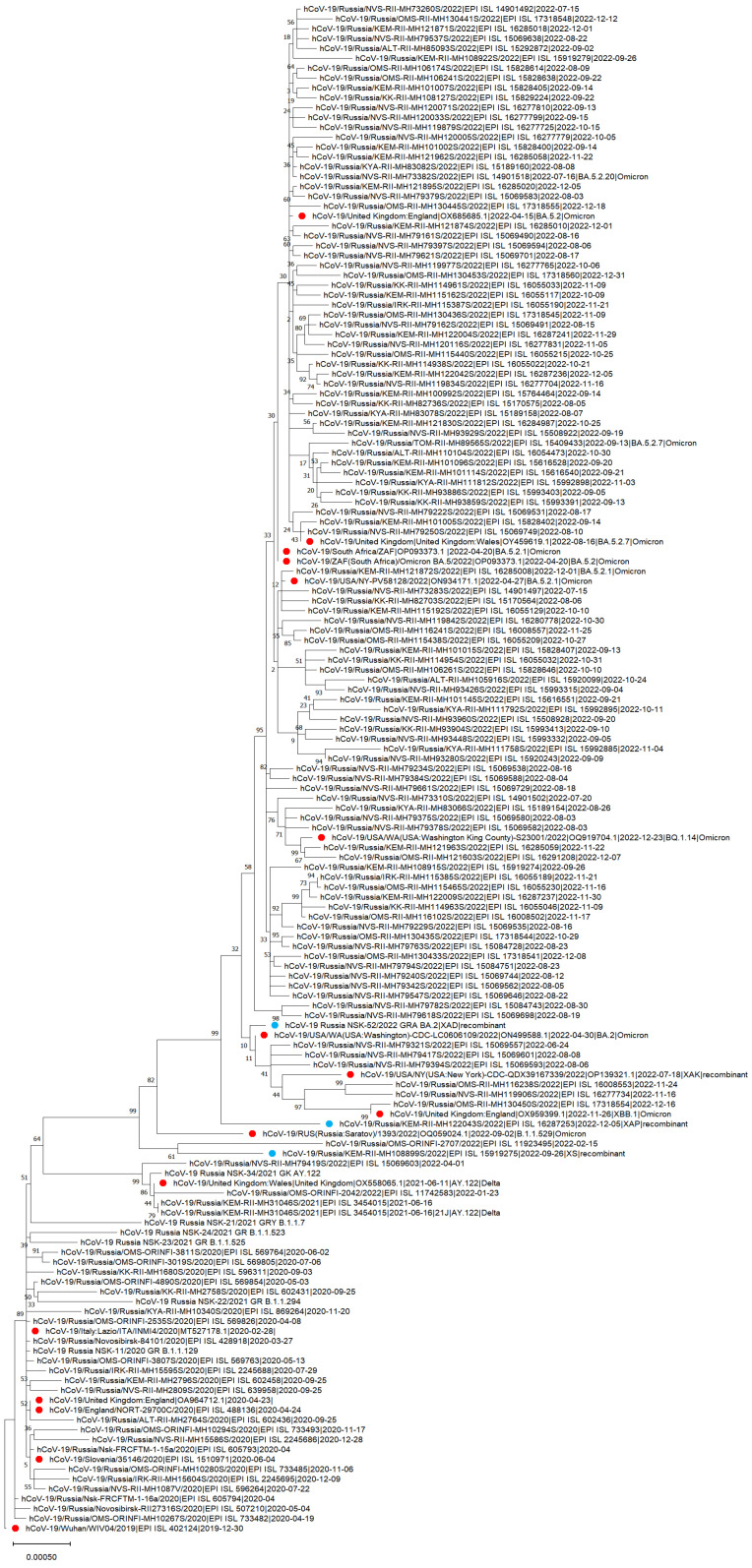
The phylogenetic dendrogram computed and visualized with MEGA11 and based on the whole genome nucleotide sequences of SARS-CoV-2 in the Siberian Federal District (SFD) in 2020–2022. Round label: red—reference sequences, blue—recombinants from SFD. We used MEGA11 [25] software for phylogenetic analysis with the Maximum Likelihood method with bootstrap support (500 replications) and utilizing a GTR + G + I substitution model.

To build a phylogenetic tree, high quality complete genomes were selected from the sample. After the removal of low-quality sequences, 906 sequences remaining were used for phylogenetic analysis. This is quite a lot and the tree would have turned out to be unreadable, so for better clarity, the final tree included only the earliest and sometimes the latest representatives of each clade for each region. The dendrogram shows the distribution of variants between regions, as well as how long some of them circulated (Figure 7). Thus, the group of variants B.1.1 (20B) remained dominant for two waves and competed with Delta. Among the variants of Omicron, BA.5.2 (22B) turned out to be the most widespread variant. It turned out to be represented in the maximum number of regions, while the other options were less common.

## 4. Discussion

The description of epidemiological processes is of fundamental importance both for epidemiology, virology, genetics and social sciences. One of the features of the current pandemic is a large number of asymptomatic carriers of the infection [6,16] actively spreading the virus among a wide range of people because the carrier, unlike a sick person, does not reduce their social activity. Timely measures taken to limit contacts and travel between regions have significantly reduced the peak incidence in the beginning of the pandemic [2,28].

In Russia, European descendants of the Wuhan strain were dominant in the first wave of incidence [15], its subsequent genovariants in wave 2, then AY.122 in the third and fourth, and Omicron in the fifth and sixth [27,28]. The variety of SARS-CoV-2 variants we have considered shows that, due to restrictions, not all possible variants entered the Siberian Federal District, while many variants of SARS-CoV-2 circulated in Russia. For example, we detected only two sequences that belonged to the Alpha variant.

In the Siberian Federal District, as in Novosibirsk [16], the beginning of the first wave lagged behind the European part of Russia. In the first three waves, the federal districts were involved in the epidemic more slowly than in the fourth and, especially, in the fifth wave, when the rise in incidence was rapid, the next week after Moscow, and simultaneously in all federal districts [28] and the world. However, when the restrictions were reduced, the beginning of the next wave began to coincide in neighboring regions, albeit with some peculiarities. We see that the introduction of restrictions effectively slows down the spread of the infection; however, social processes such as tourism, the beginning of the school year or offline communications on holidays can significantly affect the course of the epidemiological process [31].

The need for restrictions was most clearly manifested in the third and fourth waves. The Delta genovariant spread throughout the country in the second half of April 2021 and prevailed until January 2022 [28]. The morbidity situation was so catastrophic that the minimum number of sequences corresponding to the entire period of Delta dominance was deposited to GenBank and GISAID. Based on the genetic data, we assume that the appearance of the fourth wave of COVID-19 incidence in the Siberian Federal District could have been prevented, as happened in Tyva, since the third and fourth waves of incidence were caused by the same strain and the third wave began to decline. If the school year started remotely, and the restrictions continued, this could significantly slow down the spread of the Delta and the third wave would have continued to fade. Instead, educational institutions opened when the number of cases in the community was still quite high. As we know that Omicron displaced Delta very quickly [27,28], maintaining restrictions until Omicron arrived in the region could have saved a huge number of lives. The incidence would still be very high, but the people who died from the Delta could survive the Omicron as it is less lethal [30].

If we consider the incidence in the world, then in the 2019–2021 period there was no simultaneous increase or decrease even in neighboring countries [29]. In each country, under the influence of restrictions and circulating strains, its own incidence structure was formed. However, by the beginning of 2022, a sharp rise in the incidence began in most countries at the same time. This was facilitated, on the one hand, by the restrictions removed everywhere, and, on the other hand, by the high contagiousness of Omicron [30]. During the circulation period of the Omicron variant, mortality from COVID-19 dropped significantly, despite a huge rise in incidence. Continuous monitoring of circulating variants in neighboring areas would contribute to a prompt response to the changing epidemiological situation. We have identified three recombinants that appeared in densely populated areas, which is a rare event [32]. Thus, here we show the importance of sequencing even in remote regions, especially with a high population density, since the appearance of recombinants and Variants of Concern (VOCs) can go unnoticed for a long time, and late measures lead to unjustified losses. In addition, the accumulated large volumes of sequencing data make it possible to predict the evolution of viruses [32].

## 5. Conclusions

The epidemic process of a new coronavirus infection was wave-like, but not seasonal. The new wave began mainly due to the emergence and circulation among the population of new variants and sub-variants of the pathogen. The monthly trend of incidence and mortality, when compared across regions, most often repeats the weekly dynamics; however, there are also fundamental differences in some cases. The most important differences in the ten regions of the Siberian Federal District are manifested in the timing and duration of the waves, their amplitude, as well as in the mortality rate and the burden on the healthcare system. The introduced restrictions played a significant role in the pandemic processing in the Siberian Federal District; however, since the introduction and lifting of restrictions in each region did not always occur simultaneously, some regions managed to achieve a more favorable epidemiological situation, while in others the incidence grew catastrophically. The population density does not affect the incidence and mortality, but has contributed to the emergence of recombinant variants.

## Figures and Tables

**Table 1:** The number of detected infections, deaths, mortality and population of the region, as well as the maximum number of infections and deaths in one day (data for the entire analysis period: 2020–2022).

Siberian Federal District	Population Size	Population Density	Cases	Cases per 100,000	Deaths	Mortality, %	Cases Max per Day	Deaths Max per Day
Krasnoyarsk Krai	2,849,169	1.2	427,197	14,993.74	11,227	2.63	8192	39
Novosibirsk Oblast	2,780,292	15.74	310,720	11,175.8	5749	1.85	5824	18
Kemerovo Oblast	2,604,272	28.3	213,336	8191.771	2862	1.34	2585	12
Irkutsk Oblast	2,357,134	3.05	358,369	15,203.59	8685	2.42	3715	42
Altai Krai	2,268,179	14.1	316,416	13,950.22	8853	2.8	4817	32
Omsk Oblast	1,879,548	13.12	231,764	12,330.84	4567	1.97	2668	17
Tomsk Oblast	1,068,304	3.4	158,808	14,865.43	963	0.61	1673	8
Republic of Khakassia	528,338	8.66	103,230	19,538.63	2427	2.35	1202	17
Republic of Tuva	332,609	1.9	52,384	15,749.42	358	0.68	586	10
Altai Republic	221,559	2.27	40,066	18,083.67	630	1.57	306	8

**Table 2:** The diversity of genetic variants of SARS-CoV-2 in the Siberian Federal District (data for the entire analysis period: 2020–2022).

Wave	First	Second	Third	Fourth	Fifth	Sixth	
Date	15 March 2020–31 August 2020	1 September 2020–31 May 2021	1 June 2021–13 September 2021	14 September 2021–12 January 2022	13 January 2022–12 July 2022	24 June 2022–1 January 2023	
Number of Sequences	174	73	16	8	13	1605	
Dominating clade	B.1.1 (20B)	B.1.1 (20B)	AY.122 (21J) Delta	AY.122 (21J) Delta	BA.1.1 (21K) Omicron	BA.5.2 (22B) Omicron	
Number of Sequences	124 (71.3%)	46 (63%)	12 (75%)	8 (100%)	5 (38.5%)	988 (61.5%)	
Also found	B.1 (20A), B.1.1.317 (20B), B.1.1.294 (20B), B.1.1.348 (20B), B.1.1.163 (20B), B.1.1.83 (20B), B.1.1.141 (20B)	B.1.1.141 (20B) B.1.1.294 (20B) B.1.1.317 (20B) B.1.1.348 (20B) B.1.1.397 (20B) B.1.1.398 (20B) B.1.1.525 (20B) B.1.1.523 (20B) AT.1 (20B) B.1.1.7 (20I)—Alpha	B.1.1 (20B)	no	AY.126 (21J) AY.122 (21J) BA.1.15 (21K) BA.2 (21L)	BA.1 (21K) BA.1.1 (21K) BA.2 (21L) BA.2.37 (21L) BA.2.37 (21L) BA.2.38.1 (21L) BA.2.40.1 (21L) BA.2.75.2 (22D) BA.2.75.2 (22D) BA.4.4 (22A) BA.4.5 (22A) BA.4.6 (22A) BA.5 (22B) BA.5.1 (22B) BA.5.1.10 (22B) BA.5.1.12 (22B) BA.5.1.19 (22B) BA.5.1.29 (22B) BA.5.1.3 (22B) BA.5.1.30 (22B) BA.5.1.5 (22B) BA.5.2 (22B) BA.5.2.1 (22B) BA.5.2.12 (22B) BA.5.2.16 (22B) BA.5.2.20 (22B) BA.5.2.21 (22B) BA.5.2.21 (22B) BA.5.2.24 (22B) BA.5.2.27 (22B) BA.5.2.28 (22B) BA.5.2.31 (22B) BA.5.2.32 (22B) BA.5.2.32 (22B) BA.5.2.33 (22B)	BA.5.2.41 (22B) BA.5.2.44 (22B) BA.5.2.48 (22B) BA.5.2.52 (22B) BA.5.2.54 (22B) BA.5.2.56 (22B) BA.5.2.6 (22B) BA.5.2.60 (22B) BA.5.2.62 (22B) BA.5.2.7 (22B) BA.5.3.1 (22B) BA.5.5 (22B) BA.5.6 (22B) BE.1 (22B) BE.1.1 (22B) BE.1.1.2 (22B) BF.11.5 (22B) BF.28 (22B) BF.36 (22B) BF.5 (22B) BF.7 (22B) BN.1.3 (22D) BN.1.3.1 (22D) BQ.1.1 (22E) BQ.1.2.1 (22E) BQ.1.23 (22E) CK.1 (22B) CL.1.2 (22B) CT.1 (22B) XAD (recombinant) XAP (recombinant) XBB (22F) XBB.1 (22F) XBB.1.25 (22F) XS (recombinant)

**Table 3:** The recombinant genetic variants of SARS-CoV-2 in the Siberian Federal District (data for the entire analysis period: 2020–2022).

	SeqName	Nextclade–Pango	Clade	Region
1	hCoV-19_Russia_NSK-52/2022_GRA_BA.2 (not deposited)	XAD	recombinant	Novosibirsk Oblast
2	hCoV-19/Russia/KEM-RII-MH108899S/2022|EPI_ISL_15919275|2022-09-26	XS	recombinant	Kemerovo Oblast
3	hCoV-19/Russia/KEM-RII-MH122043S/2022|EPI_ISL_16287253|2022-12-05	XAP	recombinant	Kemerovo Oblast

## Data Availability

The sequence data presented in this study are openly available in the GISAID database.
